# Chronic Abdominal Pain in Median Arcuate Ligament Syndrome: A Diagnostic and Therapeutic Challenge

**DOI:** 10.7759/cureus.82309

**Published:** 2025-04-15

**Authors:** Rahul Borra, Jaafar A Hamdan, Saeid Davani, Venkat Bhaskara

**Affiliations:** 1 Internal Medicine, HCA | University of South Florida Morsani College of Medicine Graduate Medical Education (GME) Oak Hill Hospital, Brooksville, USA; 2 Dr. Kiran C. Patel College of Osteopathic Medicine, Nova Southeastern University, Fort Lauderdale, USA

**Keywords:** celiac artery variations, chronic abdominal pain, color flow doppler ultrasound, diaphragmatic crura, nausea and vomiting

## Abstract

Median arcuate ligament syndrome is a rare and difficult-to-diagnose condition that typically presents with nonspecific symptoms of abdominal pain, nausea, and vomiting. The condition is caused by compression of the celiac artery by the median arcuate ligament at the level of the diaphragmatic aortic hiatus. In this case report, we present a 25-year-old male who presented with a chief complaint of persistent abdominal pain, nausea, and vomiting. The patient’s symptoms, computed tomography imaging, and abdominal Doppler ultrasound results were consistent with the classical presentation of the disease. The patient’s symptoms definitively resolved after laparoscopic median arcuate ligament decompression.

## Introduction

Median arcuate ligament syndrome (MALS), otherwise known as Dunbar syndrome, celiac artery compression syndrome, or vascular compression syndrome [1], is a rare condition that develops secondary to an uncommon anatomical variant of the median arcuate ligament of the diaphragm. Affecting only approximately every 2 out of 100,000 patients [2], with a higher preponderance in young, thin females (F: M ratio of 4: 1)[3]. The condition occurs due to a variation in the positioning of the median arcuate ligament, a fibrous band that connects the left and right diaphragmatic crura to form the anterior aspect of the diaphragmatic aortic hiatus [4]. The constriction at this location causes external compression of both the celiac artery and the celiac plexus. The celiac artery gives rise to the left gastric, splenic, and hepatic arteries, which combined supply the stomach, liver, spleen, pancreas, gall bladder, and abdominal esophagus [5]. Due to constriction at the level of the celiac artery and celiac plexus, blood supply and neural transmission to these abdominal viscera are altered, leading to the characteristic symptoms of abdominal pain, nausea, vomiting, and weight loss [2]. Typically, the symptoms are worse on exhalation due to the caudal movement of the diaphragm and associated increased compression.

Due to the nonspecific symptoms that patients present with, the presence of MALS in patients may not be immediately apparent. Imaging plays a crucial role in obtaining the diagnosis. One of the most utilized imaging modalities for diagnosing MALS is computed tomography angiography (CTA) of the abdomen and pelvis, as this modality allows for three-dimensional visualization of the compressed celiac artery [1]. CTA has a high resolution and can show changes like post-stenotic dilatation; however, it involves ionization radiation and requires contrast, which can be a limitation in patients with renal dysfunction. Magnetic resonance angiography (MRA) can be an alternative modality for those patients who have an allergy to intravenous contrast agents. Additionally, MRA does not use ionizing radiation. Doppler ultrasonography can also be utilized due to its lack of ionizing radiation; however, a drawback of ultrasound is that it is user-dependent. The gold standard of diagnosis however is conventional angiography. To differentiate between intrinsic decreased blood flow to abdominal organs due to atherosclerosis of the celiac artery versus external compression by the median arcuate ligament, healthcare practitioners should look out for the “hook sign”, in which external compression causes the celiac artery to appear as a hook when viewed in the sagittal plane. Typically, the degree of upward deflection seen positively correlates with the degree of external compression [4]. In addition to the use of CTA, Doppler ultrasound can also be utilized to determine the degree of stenosis. With a high sensitivity and specificity of 75% and 89%, respectively [6], Doppler is useful to determine arterial flow velocities. In unaffected individuals, the peak systolic velocity (PSV) in the celiac trunk is typically between 98 and 105 cm/s [7]. However, the PSV in individuals with MALS is typically 200 cm/s or greater [8]. MRA is also valuable to the diagnosis and superior to Doppler in certain instances, particularly in regard to preoperative planning [4].

When the diagnosis of MALS is confirmed through various imaging modalities, the definitive treatment is a surgical intervention to decompress the celiac artery. Open and minimally invasive/robotic-assisted surgery can be done to divide the overlying median arcuate ligament to restore blood flow to the abdominal viscera. Furthermore, in patients who are also affected by compression of the celiac plexus, neurolysis of the plexus can also be done in order to help with pain relief [4]. In addition to decompression, celiac artery bypass, celiac artery stenting, and celiac ablation may be alternative or additional procedures that are done on a case-by-case basis [4]. Like any invasive procedure, potential complications may arise during or after surgical intervention. In individuals surgically treated for MALS, celiac artery bleeding, gastric artery bleeding, phrenic artery laceration, and aortic puncture may occur, in addition to other complications [4].

## Case presentation

Our patient was a 25-year-old male with no significant past medical history who presented with a chief complaint of three weeks of abdominal pain with associated nausea and vomiting. Per the patient’s mother who was at bedside during initial evaluation, the patient had experienced chronic abdominal pain for the last 6 years. At the time of the interview, he stated that the pain was primarily located in the left lower quadrant and “throbbing” in quality. Using the numerical ratings scale (NRS), the patient rated the pain 7/10 in severity and non-radiating. He stated that he was previously told that the pain could be secondary to biliary colic, despite the pain’s location in the left lower quadrant. He had previously elected to undergo elective cholecystectomy in 2018, with unsuccessful pain remission. He also noted at the initial presentation that he used marijuana daily and that there was no temporal relation between smoking and the occurrence of pain. Another review of social history revealed that the patient had played football during his adolescence.

**Figure 1:**
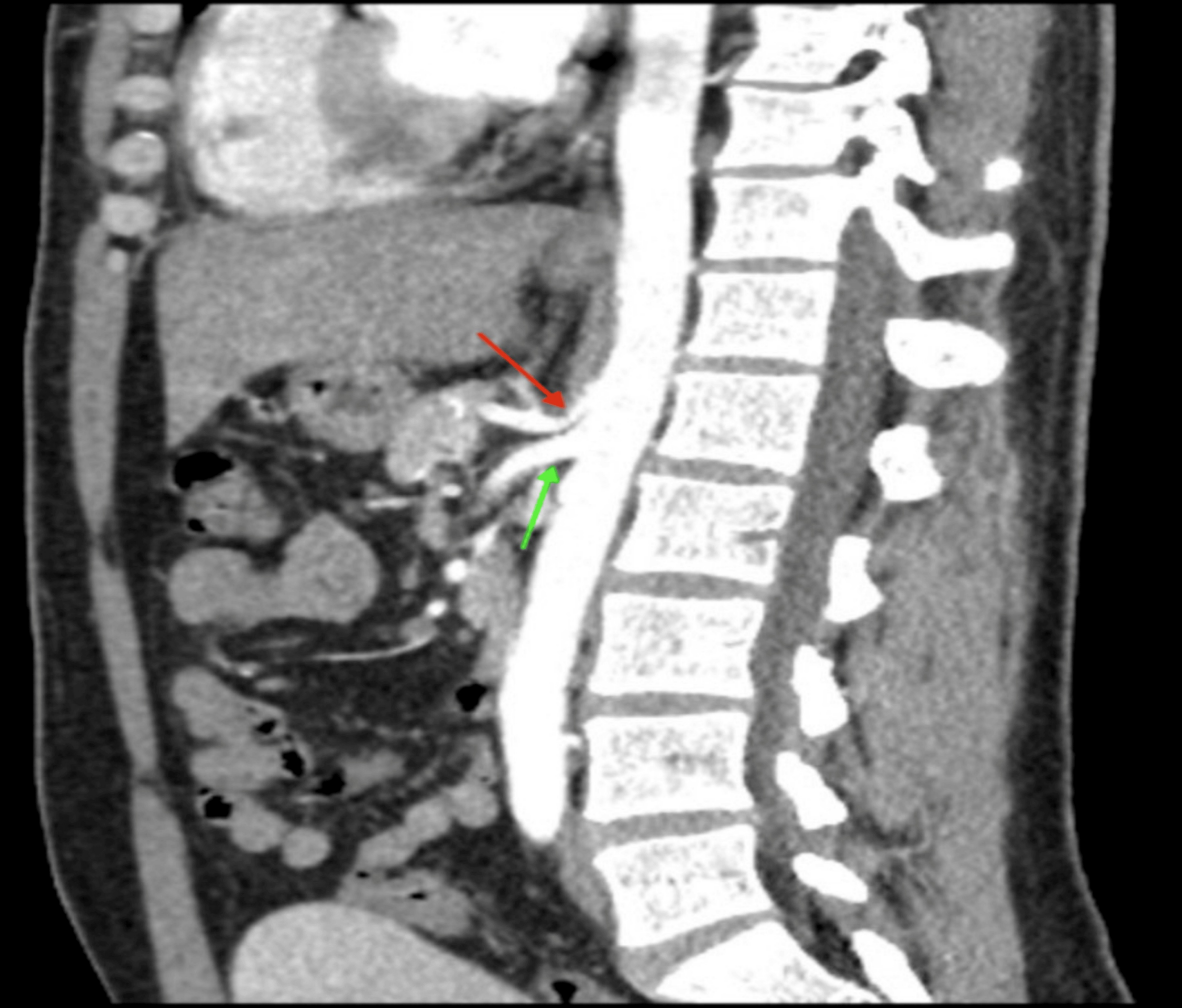
Sagittal Plane View of CTA With Contrast Revealing Stenotic Celiac Artery With Hook Sign (Red Arrow). SMA Appears Patent (Green Arrow) CTA: computed tomography angiography; SMA: superior mesenteric artery

Initial focused gastrointestinal examination revealed no scars or lesions, no visible pulsatile masses, hypoactive bowel sounds throughout the abdomen, and mild tenderness to palpation in the left lower quadrant. Initial lab work was significant for a leukocytosis of 21,300 cells/μL (normal range: 4000 - 10,500 cells/μL) and lactic acidosis of 2.3 mmol/L (normal range 0.4 - 2 mmol/L). As part of the initial evaluation, the patient underwent a CTA Abdomen/Pelvis, which revealed 60-70% stenosis at the origin of the celiac trunk and the associated “hook sign” (Figure 1) and no evidence of occlusion or abdominal aortic aneurysm, as well as nonspecific wall thickening of the sigmoid colon that indicated possible colitis; at its largest point, the bowel wall thickness was noted to be 1.4 cm. IV morphine 4 mg q6h as needed was started for pain control. Due to the findings on CT, general surgery and vascular surgery were consulted for further treatment recommendations for sigmoid colitis and celiac artery stenosis. 

The patient was seen by vascular surgery on the second day of admission to the hospital. When speaking to the vascular surgery team, he did note that certain foods tended to exacerbate his symptoms, although overall the symptoms occurred at random. He also stated that cessation of marijuana use for a 6-month period had no effect on the level or frequency of the pain. General surgery also examined the patient on day 2 and believed the symptoms were in fact due to celiac artery stenosis. Doppler ultrasound of the aorta was ordered to determine arterial velocities of the mesenteric vessels. Gastroenterology was also consulted per general surgery recommendations.

**Figure 2:**
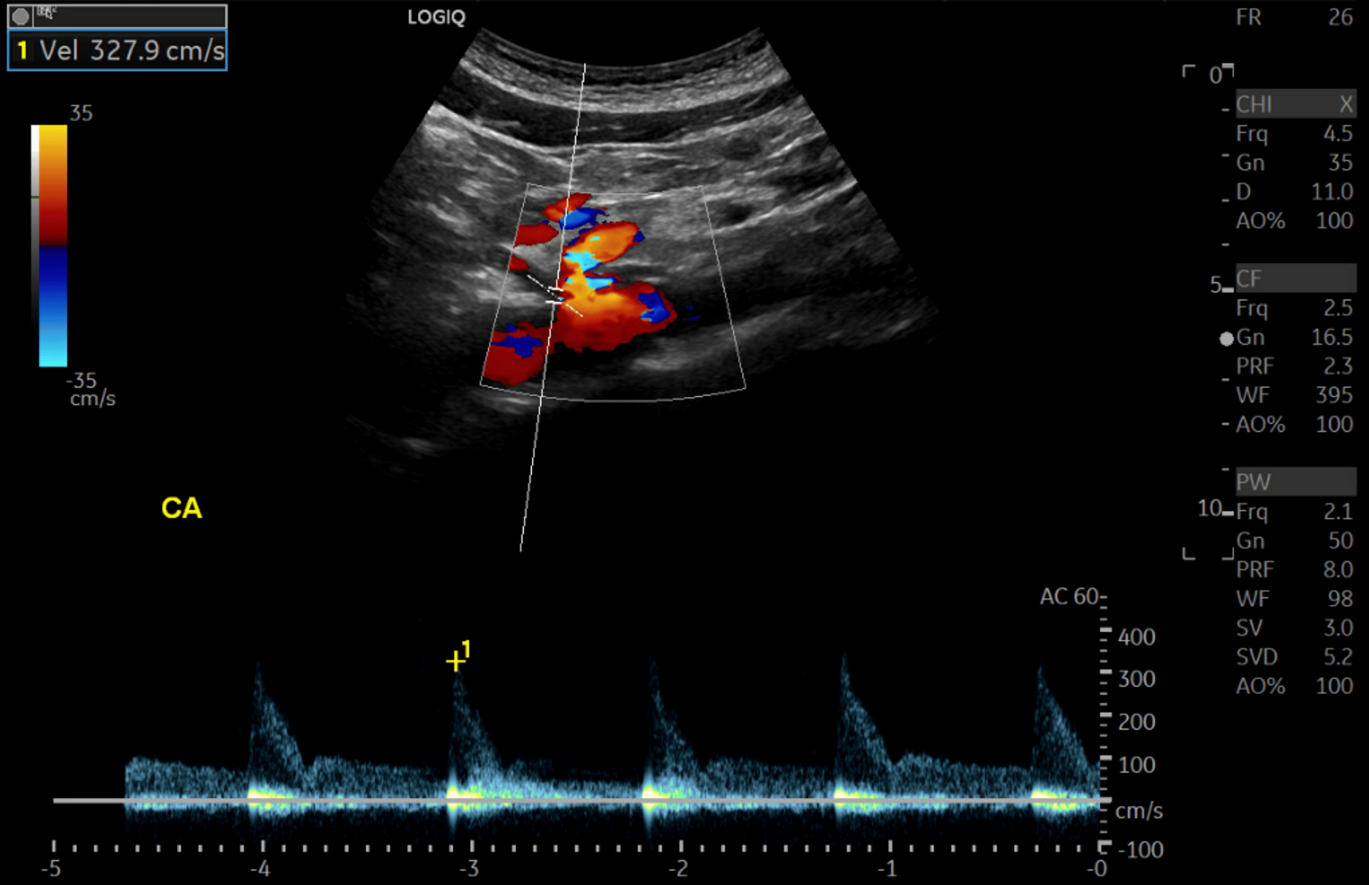
Flow velocity within the celiac artery shows flow at 327.2 cm/s.

On the third day of hospitalization, an ultrasound of the aorta with flow velocities revealed flow in the celiac artery to be elevated at 327.9 cm/s (normal: 98 - 105 cm/s) (Figure 2). Flow velocities in the superior mesenteric artery, right renal artery, and left renal artery were all within the normal range at 195.6 cm/s, 74.2 cm/s, and 74.7 cm/s, respectively. The gastroenterology team saw the patient and ordered an upper GI series to evaluate the esophagus, stomach, and duodenum; imaging revealed no abnormalities. In addition, GI believed the patient would benefit from esophagogastroduodenoscopy (EGD) and colonoscopy to rule out other pathologies that could be contributing. EGD and colonoscopy done the same day were normal as well.

By the fourth day of hospitalization, it was determined that the patient’s symptoms were solely explained by celiac artery stenosis. This diagnosis was supported by the increased flow velocities in the celiac artery, which was nearly more than triple the upper limit of normal. At this time, further investigation revealed that the patient had a gradual progression of the narrowing of the celiac artery over the previous 10 years. When compared to a CT abdomen/pelvis in 2013, a CT abdomen done in 2023 revealed a significant decline in celiac artery diameter when seen on a sagittal section. Imaging from 2013 revealed a celiac artery diameter of 7.4 mm, while imaging done during this admission showed a diameter of 5.5 mm, a reduction of 1.9 mm. Further evidence of decreased perfusion to the mesentery was supported by irregularities in perfusion to the spleen on the CT abdomen done in 2023. The patient was scheduled and taken for the robotic-assisted release of the median arcuate ligament. Over the course of the next day, the patient’s symptoms completely subsided, he was able to tolerate a regular diet with no nausea or vomiting, and he was subsequently discharged with instructions to follow up with the surgery team on an outpatient basis.

## Discussion

MALS also called Dunbar syndrome is a vascular compression syndrome. Also technically known as celiac artery compression syndrome, it results from the compression of the celiac axis by the median arcuate ligament of the diaphragmatic crura. It was first described anatomically in 1917 by Lipshutz, who performed cadaveric dissections and showed the overlapping of the celiac artery by the diaphragmatic crura leading to compression [9]. Following this, Harjola reported a case of symptomatic relief of postprandial epigastric pain in a 57-year-old man after surgical decompression of the celiac artery from fibrosis of the celiac ganglion [10]. Following this, in 1965, Dunbar et al. reported a case series regarding various surgical techniques for the management of patients with MALS. [11]

MALS, being a rare condition, is a diagnosis of exclusion. In the course of diagnosing the disease, more common etiologies of unexplained abdominal pain and nausea must first be ruled out before the diagnosis of MALS can be made. Due to the condition being a structural rather than functional disease, surgical intervention continues to remain the definitive treatment option.

Multiple imaging modalities are currently available to assist in the diagnosis of MALS, most notably CTA and Doppler ultrasound. Imaging via CTA may reveal the characteristic “hook sign” caused by external compression of the celiac artery. In situations where Doppler ultrasound is utilized, increased flow through the celiac artery further indicates a diagnosis of MALS. If not diagnosed and treated in a timely fashion, untreated MALS may lead to potentially serious sequelae, such as arterial aneurysms. If present, these arterial aneurysms primarily exist in the collateral vessels that develop via the superior mesenteric artery. Once these aneurysms are developed, treatment may require embolization of these vessels, either via stenting or coiling [2]. Interestingly enough, the collateral vessels that form the aneurysms may also be the reason that the symptoms of MALS may take years to present, as increased blood flow to the foregut provided by these collaterals plays a role in delaying the characteristic abdominal pain and nausea that define the disease.

To definitively treat this condition, surgical decompression with release of the median arcuate ligament is performed. In certain situations, a celiac plexus block can be done alongside the surgical decompression. In the past, the procedure was traditionally done with an open approach; however, with the current technological advancements, the procedure is typically now done either laparoscopically or robotically. In conjunction with surgical management, newer techniques via interventional radiology, such as percutaneous transluminal angioplasty (PTA), may be used, although this method should not be used by itself as it does not address the underlying celiac trunk compression [2].In regards to treating the celiac plexus, it has been shown that combining both decompression of the median arcuate ligament along celiac artery dissection improves postoperative outcomes compared to decompression alone. Like any surgical procedure, however, decompression is not without potential complications. The most concerning complication of decompression that surgeons should be vigilant for is injury to the abdominal aorta, as this may significantly increase the patient’s morbidity and mortality [12].

Even though MALS is typically attributed to a structural abnormality of the diaphragm, there are other factors to consider. Abdominal manipulation via surgeries has been shown to play a role in the development of this condition. Aside from surgical contributions to the development of this condition, perhaps more studies should be done on the role of repetitive micro-trauma via contact sports in this condition’s development, particularly in the younger populations.

## Conclusions

MALS is a rare entity and a diagnosis of exclusion. The diagnosis is difficult and needs a high grade of suspicion with supportive imaging findings. Its clinical suspicion arises when usual medical conditions causing abdominal pain or other digestive symptoms are ruled out. Duplex ultrasonography (DUS) and CT abdominal angiography are the most used imaging techniques in the current day when MALS is highly suspected. MALS treatment is based on celiac trunk decompression with celiac lymphadenectomy, followed, in specific cases, by a percutaneous transluminal angioplasty. This surgical procedure can be done either through an open surgical approach, laparoscopic approach, or robotic-assisted routes. Vascular reconstruction of the celiac trunk and celiac neurolysis also remains a solution in sectioned cases.
